# Worse survival despite indolent features for triple-negative invasive lobular carcinoma: a Swedish nationwide registry-based study

**DOI:** 10.1007/s10549-025-07862-9

**Published:** 2025-11-21

**Authors:** Jenny Nyqvist-Streng, Chaido Chamalidou, Anikó Kovacs, Toshima Z. Parris

**Affiliations:** 1 Region Västra Götaland, Department of Surgery, Skaraborg Hospital, Skövde, Sweden; 2 Department of Surgery, Institute of Clinical Sciences, Sahlgrenska Academy, University of Gothenburg, Gothenburg, Sweden; 3 Department of Oncology, Institute of Clinical Sciences, Sahlgrenska Academy, University of Gothenburg, Gothenburg, Sweden; 4 Region Västra Götaland, Department of Oncology, Skaraborg Hospital, Skövde, Sweden; 5 Region Västra Götaland, Department of Clinical Pathology, Sahlgrenska University Hospital, Gothenburg, Sweden; 6 Sahlgrenska Center for Cancer Research, Sahlgrenska Academy, University of Gothenburg, Gothenburg, Sweden

**Keywords:** Histological subtype, Invasive breast carcinoma, Sociodemographic factors, Machine learning, Prognosis, Survival outcomes, Metastatic pattern, Marital status

## Abstract

**Purpose:**

To evaluate differences in clinical outcomes, treatments received, recurrence, and sociodemographic characteristics in patients with triple-negative breast cancer (TNBC) classified as invasive lobular carcinoma (TNBC–ILC) or invasive carcinoma of no special type (TNBC–NST).

**Methods:**

Using national registry data, we conducted a retrospective, population-based cohort study of 6449 women diagnosed with primary TNBC (stratified by histological subtype) in Sweden (2007–2021). Clinical and treatment data were analyzed using descriptive statistics, logistic regression, machine learning (Boruta/XGBoost), and Cox proportional hazards models adjusted for patient age, tumor size, grade, nodal status, comorbidities, and receipt of adjuvant chemotherapy (ACT).

**Results:**

TNBC–ILC accounted for 2.7% of all TNBC cases and affected older patients (median age 70 vs 62 years). Compared to TNBC–NST, TNBC–ILC had lower Ki-67, fewer high-grade tumors, higher T stage, and greater socioeconomic vulnerability. Machine learning identified age and post-operative tumor size as key predictive features of TNBC–ILC. ACT was administered to 40% of TNBC–ILC versus 59% of TNBC–NST cases (P < 0.001), with a survival benefit observed only in TNBC–NST. TNBC–ILC patients aged 50–64 years were less likely to receive ACT. Despite lower proliferative activity, TNBC–ILC was associated with worse overall (OS; adj-HR 1.39, 95% CI 1.04–1.86) and disease-specific survival (DSS; adj-HR 1.98, 95% CI 1.41–2.79), particularly in patients ≥ 50 years of age. TNBC–ILC patients ≥ 75 years had the poorest 5-year survival (DSS 55%; OS 42%).

**Conclusions:**

TNBC–ILC is a distinct subgroup with older age, lower grade and Ki-67, undertreatment, and poorer survival, emphasizing the need for age- and subtype-specific treatment strategies.

**Supplementary Information:**

The online version contains supplementary material available at 10.1007/s10549-025-07862-9.

## Background

Of the approximately 2.3 million breast cancer cases diagnosed worldwide each year, invasive lobular carcinomas (ILC; 5–15%) represent the second most common histologic subtype, following invasive breast carcinoma of no special type (NST, also known as invasive ductal carcinoma; 80%) [1, 2]. Compared to NST, ILC is more challenging to detect with physical exams or mammography due to its characteristic discohesive growth patterns, in which tumor cells infiltrate the fibrous stroma in single files rather than forming cohesive clumps [3]. ILC incidence rates are on the rise, likely attributed to widespread use of hormone replacement therapy and advances in diagnostic methods [4–9]. ILC is also more frequently associated with older age at diagnosis, late-stage disease, bilateral involvement, and risk of metastasis to the peritoneum, gastrointestinal tract, and leptomeninges [4, 5, 10–14]. Findings from a large Swedish population-based cohort with two decades of follow-up further highlighted the distinct survival patterns of ILC, demonstrating improved outcomes compared to NST during the first five years after diagnosis but a significantly increased excess mortality 10–15 years later, consistent with the propensity for late recurrences in ILC [15]. Moreover, biological and clinical differences between ILC and NST are subtype-dependent: ILC is more frequently hormone receptor-positive and less commonly triple-negative breast cancer (TNBC), contributing to divergent patterns of disease behavior and clinical outcomesDIN EN.CITE [15].

Diagnostic criteria guiding breast cancer treatment have progressively shifted from a reliance on histopathological evaluation to prioritizing receptor status and gene expression profiling, highlighting the integration of molecular subtyping into clinical decision-making [16, 17]. Notably, ILC demonstrates lower responsiveness to both neoadjuvant and adjuvant chemotherapy compared to NST, which remains the standard adjuvant treatment for TNBC [18]. While the histologic distinction between ILC and NST is generally straightforward, a recent study further characterized ILC into a subgroup of low- to intermediate histological grade breast carcinomas with lobular growth features. This entity, termed lobular-like invasive mammary carcinomas (LLIMCas), is characterized by E-cadherin and p120 immunopositivity and an absence of CDH1 loss-of-function mutations, which are common in classic ILCs [3]. Subtyping of invasive lobular carcinoma is still challenging for pathologists when it is not the classical type with its monomorphic cells arranged in an Indian file. There are already AI tools available that can classify invasive lobular carcinoma into its less common subgroups, such as the alveolar, histiocytoid, signet ring cell type, ILC with mucin, ILC with tubular elements, ILC with neuroendocrine features and pleomorphic types [19–23].

Though relatively rare, a diagnosis of lobular TNBC (TNBC–ILC; 0.9–2.3% of all ILC cases) may indicate a substantially poorer prognosis compared to TNBC of no special type (TNBC–NST; 10–12.5% of all NST cases) [24–30]. Recent data from the National Cancer Database showed that women with TNBC–ILC were typically older at diagnosis and significantly less likely to achieve a complete pathological response to neoadjuvant chemotherapy [18]. Although previous studies also suggest that TNBC–ILC may differ significantly from TNBC–NST in terms of age at diagnosis, tumor biology, metastatic pattern, and response to treatment, robust population-based data are limited [31]. In this retrospective, population-based cohort study, we utilized nationwide Swedish cancer registry data to provide a comprehensive comparison of patients with TNBC–ILC and TNBC–NST with regard to clinical presentation, tumor characteristics, treatment modalities, and outcomes. A total of 6449 women with primary invasive TNBC diagnosed between 2007 and 2021 were included after exclusion of mixed histology and early deaths, thereby demonstrating that TNBC–ILC is a rare entity representing a distinct subgroup compared to TNBC–NST.

## Methods

### Study population and data

**Fig. 1:**
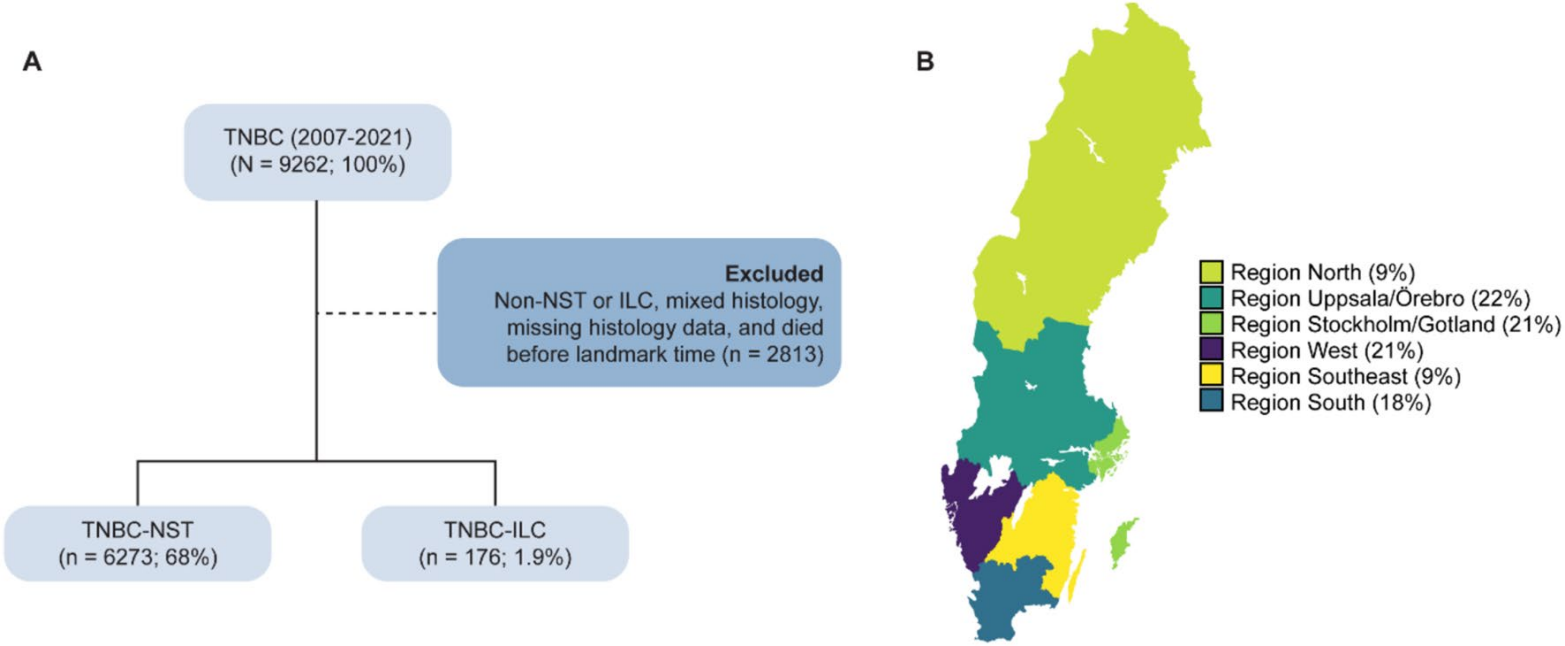
Study overview. **A** Flowchart of triple-negative breast cancer (TNBC) patient selection to include those with TNBC of no special type (TNBC–NST), TNBC invasive lobular carcinoma (TNBC–ILC), and that died after the landmark time. **B** Map of Sweden showing the percentage of patients receiving a TNBC–NST or TNBC–ILC diagnosis in the six healthcare regions included in the study

Nationwide registry-based data were retrieved from the National Breast Cancer Register (NBCR; data accessed 2023–2024), Swedish Patient Register (SPR; data accessed 2023), Swedish Cause of Death (SCDR; data accessed 2023), and Statistics Sweden (SCB; data accessed 2024) for 9262 women diagnosed with primary, invasive TNBC (ER- [estrogen receptor], PR- [progesterone receptor], and HER2- [human epidermal growth factor receptor 2] at baseline or after surgery) between 2007 and 2021 in Sweden, as described elsewhere[32]. In brief, data were compiled from NBCR for patient characteristics, primary tumor features, postoperative treatment, relapse, SCDR for date and cause of death, SPR for comorbidities, and SCB for sociodemographics. Patients with non-TNBC–NST, non-TNBC–ILC, mixed histology, and those that died before the landmark time were excluded (Fig. 1A). The landmark time was defined as 6 months after the date of diagnosis, i.e., the likely timeframe for treatment start. For bilateral breast cancer, only the first detected tumor was included. The administrative cut-off date was set to January 1, 2023. All procedures were done in accordance with the Declaration of Helsinki and approved by the Central Ethical Review Board and the Swedish Ethical Review Authority (reference numbers 2019–05676; 2021–04421; 2022–02946-02; 2023–07905-02). A patient advocate was involved to ensure that the present study aligned with patient-centered outcomes.

### Definitions

Using national Swedish guidelines and the 2017 St. Gallen molecular classification [33], ER and PR < 10% were classified as negative. HER2-negativity was defined as an immunohistochemistry score 0, 1+ or 2+ without HER2 amplification. Ki67 indices < 20% and ≥ 20% were classified as low and high expression, respectively. The weighted Charlson comorbidity index (CCIw), defined as a measure of the burden of comorbid conditions, was calculated as previously described [34] and stratified into three groups: CCIw = 0, CCIw = 1–3, and CCIw = 4–10. Data for relapse were only available for 4/6 healthcare regions in Sweden (Region Stockholm/Gotland, Region South, Region Southeast, and Region West). The time to relapse or death was classified as (1) rapid relapse or death within 2 years of initial diagnosis or (2) late relapse or death after 2 years. Total household income was classified into quartiles, ranging from the lowest (Q1) to the highest (Q4). Employment status was classified into employed (including retirees) and unemployed.

### Statistical analysis

Statistical analyses were performed using R/Bioconductor (version 4.2.2), with a significance threshold of 0.05 and two-sided P-values. Descriptive statistics were calculated for patient and tumor characteristics (e.g., age and tumor histology) using the tableone package (version 0.13.2); p-values were calculated using Chi-square tests (with continuity correction when expected counts were small) for categorical variables and ANOVA for continuous variables. Variables (“features” herein) associated with TNBC–ILC were selected using the Boruta algorithm (Boruta package version 8.0.0) with xgBoost (version 1.7.11.1) and SHAPforxgboost (version 0.1.3). Cumulative incidence curves for competing risks were constructed using ggcompetingrisks function in the survminer package (version 0.4.9). Survival probabilities for disease-specific survival (DSS; time from the landmark time to death of breast cancer) and overall survival (OS; time from the landmark time to death of any cause) were depicted with Kaplan–Meier curves and tested using the log-rank test with the ggsurvfit package (version 0.3.0). Multivariable Cox proportional hazards models were adjusted for patient age, tumor size, Nottingham histologic grade (NHG), axillary lymph node status, CCIw group, and adjuvant chemotherapy received using the survival package (version 3.4–0).

## Results

### Patient demographics

After excluding samples with mixed histology and patients that died before the landmark time, 6449 patients with TNBC were included in the study (Fig. 1 and Table 1). As expected, TNBC–NST (97%) was significantly more common than TNBC–ILC (2.7%). Nevertheless, patients with TNBC–ILC were comparatively older (median, 70 years [IQR 63, 77] vs 62 years [IQR 50, 72]) and more frequently ≥ 65 years of age (70% vs 44%). Patients with TNBC–ILC were also more likely to be widowed (23% vs 13%) and unemployed (69% vs 52%). No differences in household income or educational level were found based on tumor histology. Possibly due to their advanced age, 47% of patients with TNBC–ILC had comorbid conditions, particularly other malignancies including malignant neoplasms of the colon, lung, bone and articular cartilage, as well as malignant melanoma and unspecified malignant neoplasm of the skin. Gynecological malignancies comprised tumors of the myometrium, ovary, and fallopian tube. Additional hematologic and metastatic diseases were also present, including secondary neoplasms of the axillary and retroperitoneal lymph nodes, follicular lymphoma, and chronic lymphocytic leukemia of B-cell type.

**Table 1:** Patient demographics and comorbidity of the 6449 TNBC patients in Sweden (2007–2021), stratified by histology

	Overall (n = 6449)	TNBC–NST (n = 6273)	TNBC–ILC (n = 176)	P
Swedish region (%)				0.338
North	571 (8.9)	556 (8.9)	15 (8.5)	
Stockholm/Gotland	1357 (21.0)	1320 (21.0)	37 (21.0)	
South	1134 (17.6)	1092 (17.4)	42 (23.9)	
Southeast	611 (9.5)	594 (9.5)	17 (9.7)	
Uppsala/Örebro	1420 (22.0)	1386 (22.1)	34 (19.3)	
West	1356 (21.0)	1325 (21.1)	31 (17.6)	
Age, years (median [IQR])	62.00 [50.00, 72.00]	62.00 [50.00, 72.00]	70.00 [63.00, 77.00]	** < 0.001**
Age group, years (%)				** < 0.001**
< 40	546 (8.5)	543 (8.7)	3 (1.7)	
40–49	958 (14.9)	951 (15.2)	7 (4.0)	
50–64	2072 (32.1)	2029 (32.3)	43 (24.4)	
65–74	1551 (24.1)	1480 (23.6)	71 (40.3)	
≥ 75	1322 (20.5)	1270 (20.2)	52 (29.5)	
Marital status (%)				**0.006**
Divorced	1213 (18.8)	1191 (19.0)	22 (12.5)	
Married	3135 (48.6)	3048 (48.6)	87 (49.4)	
Registered partner	1 (0.0)	1 (0.0)	0 (0.0)	
Single	1167 (18.1)	1141 (18.2)	26 (14.8)	
Widowed	875 (13.6)	835 (13.3)	40 (22.7)	
Missing data	58 (0.9)	57 (0.9)	1 (0.6)	
Household income (%)				0.971
Q1	1590 (24.7)	1547 (24.7)	43 (24.4)	
Q2	1598 (24.8)	1554 (24.8)	44 (25.0)	
Q3	1583 (24.5)	1537 (24.5)	46 (26.1)	
Q4	1620 (25.1)	1578 (25.2)	42 (23.9)	
Missing data	58 (0.9)	57 (0.9)	1 (0.6)	
Educational level (%)				0.776
< High school	1342 (20.8)	1308 (20.9)	34 (19.3)	
High school	2703 (41.9)	2632 (42.0)	71 (40.3)	
Higher education	2301 (35.7)	2234 (35.6)	67 (38.1)	
Missing data	103 (1.6)	99 (1.6)	4 (2.3)	
Employment (%)				** < 0.001**
Employed	2970 (46.1)	2917 (46.5)	53 (30.1)	
Unemployed	3406 (52.8)	3285 (52.4)	121 (68.8)	
Missing data	73 (1.1)	71 (1.1)	2 (1.1)	
Comorbidities (%)				
Myocardial infarction	154 (2.4)	146 (2.3)	8 (4.5)	0.099
Congestive heart failure	132 (2.0)	130 (2.1)	2 (1.1)	0.552
Peripheral vascular disease	105 (1.6)	103 (1.6)	2 (1.1)	0.825
Cerebrovascular disease	349 (5.4)	336 (5.4)	13 (7.4)	0.315
Chronic obstructive pulmonary disease	117 (1.8)	113 (1.8)	4 (2.3)	0.86
Chronic other pulmonary disease	216 (3.3)	210 (3.3)	6 (3.4)	1
Rheumatic disease	292 (4.5)	278 (4.4)	14 (8.0)	0.042
Dementia	39 (0.6)	39 (0.6)	0 (0.0)	0.578
Hemiplegia	22 (0.3)	22 (0.4)	0 (0.0)	0.895
Diabetes without chronic complication	6 (0.1)	6 (0.1)	0 (0.0)	1
Diabetes with chronic complication	101 (1.6)	98 (1.6)	3 (1.7)	1
Renal disease	56 (0.9)	55 (0.9)	1 (0.6)	0.981
Mild liver disease	45 (0.7)	45 (0.7)	0 (0.0)	0.504
Liver special	4 (0.1)	4 (0.1)	0 (0.0)	1
Severe liver disease	6 (0.1)	6 (0.1)	0 (0.0)	1
Peptic ulcer disease	109 (1.7)	108 (1.7)	1 (0.6)	0.382
Malignancy	937 (14.5)	894 (14.3)	43 (24.4)	** < 0.001**
Metastatic solid tumor	57 (0.9)	53 (0.8)	4 (2.3)	0.112
AIDS	3 (0.0)	3 (0.0)	0 (0.0)	1
CCIw group (%)				** < 0.001**
CCIw = 0	4382 (67.9)	4288 (68.4)	94 (53.4)	
CCIw = 1–3	1879 (29.1)	1805 (28.8)	74 (42.0)	
CCIw = 4–10	188 (2.9)	180 (2.9)	8 (4.5)	

*CCIw* weighted Charlson comorbidity index, *Q1–Q4* Household income quartiles, *TNBC* Triple-negative breast cancer, *TNBC–ILC* Triple-negative breast cancer–invasive lobular cancer, *TNBC–NST* Triple-negative breast cancer of no special type

Statistically significant differences (p 0.05) are highlighted in bold

### Tumor features

**Fig. 2:**
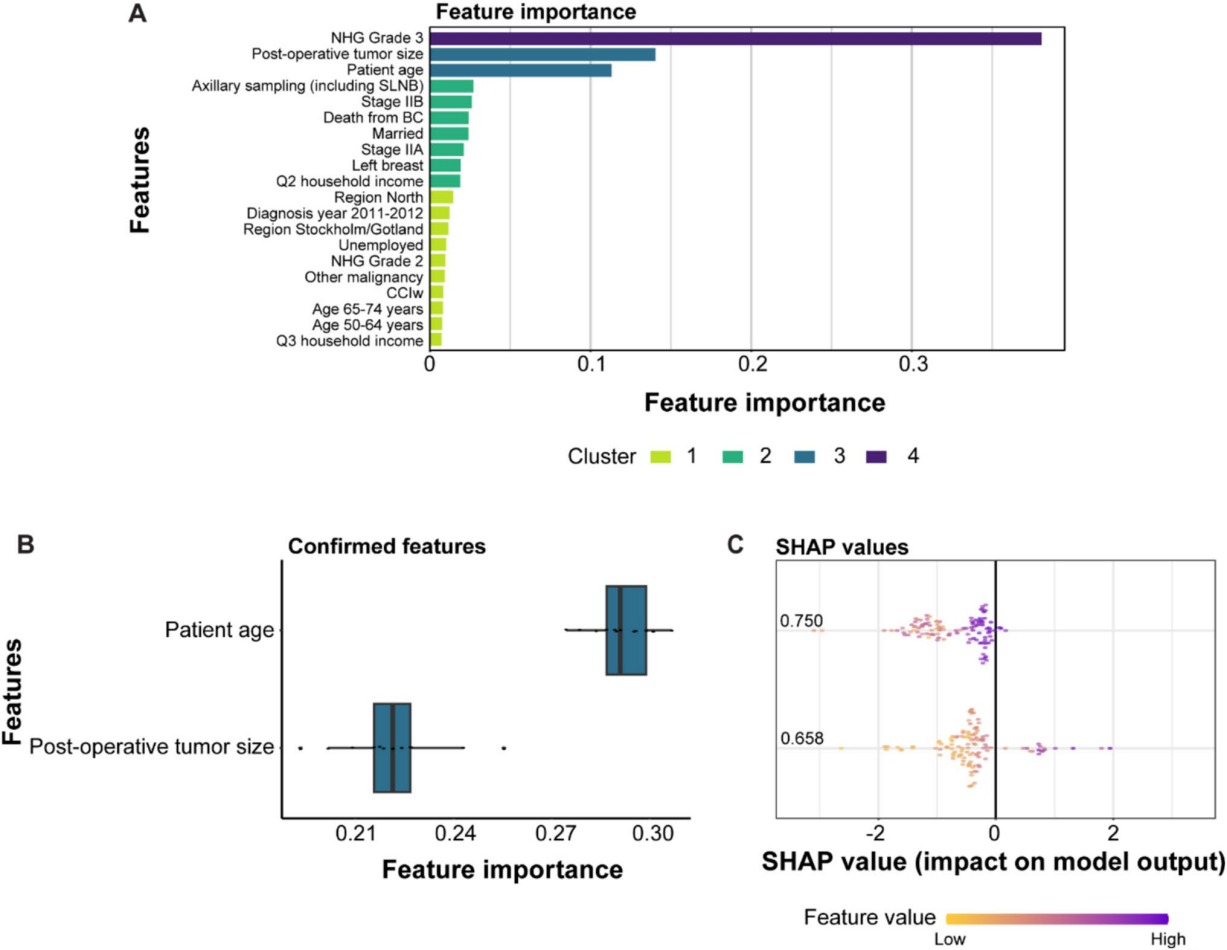
Boruta feature selection associated with triple-negative breast cancer invasive lobular carcinoma (TNBC–ILC) using xgBoost with SHAP analysis. **A** Top 20 xgBoost features with their measure of contribution for optimizing the model. **B** Confirmed features using Boruta importance evaluation. **C** SHAP values for the confirmed features

Approximately 89% of the patient cohort were stage I–II, with no differences based on histology (Table 2). TNBC–ILCs were more frequently classified as T3 (11% vs 5%) and exhibited lower proliferative activity, with low Ki67% (23% vs 58%) and fewer NHG 3 tumors (23% vs 78%) compared with TNBC–NST (Table 2). Notably, despite lower Ki67 and grade, TNBC–ILC showed similar axillary lymph node positivity rates to TNBC–NST, indicating comparable nodal aggressiveness. Boruta feature selection using xgBoost with SHAP analysis illustrated that patient age and post-operative tumor size were the most important features for identifying patients with TNBC–ILC (Fig. 2).

**Table 2:** Clinicopathological characteristics and treatment for the 6449 TNBC patients in Sweden (2007–2021), stratified by histology

	Overall (n = 6449)	TNBC–NST (n = 6273)	TNBC–ILC (n = 176)	P
Tumor size, mm (median [IQR])*	30.00 [23.00, 40.00]	30.00 [23.00, 40.00]	25.00 [19.00, 40.00]	0.406
T stage (%)*				**0.014**
T0	404 (6.3)	394 (6.3)	10 (5.7)	
T1	2940 (45.6)	2874 (45.8)	66 (37.5)	
T2	2614 (40.5)	2540 (40.5)	74 (42.0)	
T3	341 (5.3)	321 (5.1)	20 (11.4)	
T4a	5 (0.1)	5 (0.1)	0 (0.0)	
T4b	35 (0.5)	35 (0.6)	0 (0.0)	
T4d	20 (0.3)	19 (0.3)	1 (0.6)	
Tis	65 (1.0)	62 (1.0)	3 (1.7)	
TX	24 (0.4)	22 (0.4)	2 (1.1)	
Missing data	1 (0.0)	1 (0.0)	0 (0.0)	
N stage (%)*				0.738
N0	5302 (82.2)	5157 (82.2)	145 (82.4)	
N1	1046 (16.2)	1019 (16.2)	27 (15.3)	
N2	41 (0.6)	40 (0.6)	1 (0.6)	
N3	25 (0.4)	23 (0.4)	2 (1.1)	
NX	34 (0.5)	33 (0.5)	1 (0.6)	
Missing data	1 (0.0)	1 (0.0)	0 (0.0)	
Stage (%)				0.167
0	60 (0.9)	57 (0.9)	3 (1.7)	
IA	2643 (41.0)	2586 (41.2)	57 (32.4)	
IIA	2277 (35.3)	2209 (35.2)	68 (38.6)	
IIB	778 (12.1)	753 (12.0)	25 (14.2)	
IIIA	167 (2.6)	160 (2.6)	7 (4.0)	
IIIB	60 (0.9)	59 (0.9)	1 (0.6)	
IIIC	20 (0.3)	18 (0.3)	2 (1.1)	
IV	10 (0.2)	10 (0.2)	0 (0.0)	
Unspecified	434 (6.7)	421 (6.7)	13 (7.4)	
NHG (%)†				** < 0.001**
Grade 1	104 (1.6)	101 (1.6)	3 (1.7)	
Grade 2	1263 (19.6)	1137 (18.1)	126 (71.6)	
Grade 3	4953 (76.8)	4912 (78.3)	41 (23.3)	
Ki67% [mean (SD)]†	57.18 (26.98)	58.10 (26.55)	23.15 (20.22)	** < 0.001**
NACT (%)	688 (10.7)	665 (10.6)	23 (13.1)	0.172
Type of NACT (%)				0.651
Anthracycline+taxane	607 (9.4)	587 (9.4)	20 (11.4)	
Anthracycline-based	40 (0.6)	38 (0.6)	2 (1.1)	
Taxane-based	41 (0.6)	40 (0.6)	1 (0.6)	
Axillary surgery type (%)				**0.003**
SLNB	4103 (63.6)	4014 (64.0)	89 (50.6)	
AD	1315 (20.4)	1273 (20.3)	42 (23.9)	
Sampling (including SLNB)	97 (1.5)	94 (1.5)	3 (1.7)	
SLNB + AD	744 (11.5)	712 (11.4)	32 (18.2)	
Surgical treatment (%)				** < 0.001**
Only axillary surgery	8 (0.1)	8 (0.1)	0 (0.0)	
Breast-conserving surgery	3544 (55.0)	3478 (55.4)	66 (37.5)	
Mastectomy	2832 (43.9)	2722 (43.4)	110 (62.5)	
Subcutaneous mastectomy	40 (0.6)	40 (0.6)	0 (0.0)	
Missing data	25 (0.4)	25 (0.4)	0 (0.0)	
ACT (%)	3757 (58.3)	3686 (58.8)	71 (40.3)	** < 0.001**
ACT according to plan (%)	2863 (44.4)	2809 (44.8)	54 (30.7)	** < 0.001**
Type of ACT (%)				** < 0.001**
Anthracycline + taxane	1594 (24.7)	1564 (24.9)	30 (17.0)	
Anthracycline-based	1511 (23.4)	1489 (23.7)	22 (12.5)	
Taxane-based	186 (2.9)	183 (2.9)	3 (1.7)	
Other	334 (5.2)	322 (5.1)	12 (6.8)	
ART (%)	3764 (58.4)	3667 (58.5)	97 (55.1)	0.356
Adjuvant bisphosphonate therapy (%)	718 (11.1)	687 (11.0)	31 (17.6)	**0.02**
Locoregional recurrence (%)				
Local	152 (2.4)	149 (2.4)	3 (1.7)	0.744
Regional	96 (1.5)	95 (1.5)	1 (0.6)	0.48
Distant relapse (%)				**0.045**
Distant relapse	493 (7.6)	471 (7.5)	22 (12.5)	
No distant relapse	3965 (61.5)	3860 (61.5)	105 (59.7)	
Not available	1991 (30.9)	1942 (31.0)	49 (27.8)	
Distant relapse or death (%)				**0.013**
Late relapse or death	5245 (81.3)	5115 (81.5)	130 (73.9)	
Rapid relapse or death	1204 (18.7)	1158 (18.5)	46 (26.1)	

*ACT* Adjuvant chemotherapy, *AD* axillary dissection, *ART* Adjuvant radiotherapy, *NACT* Neoadjuvant chemotherapy, *NHG* Nottingham grade, *SLNB* Sentinel lymph node biopsy, *TNBC* Triple-negative breast cancer, *TNBC–ILC* Triple-negative breast cancer–invasive lobular cancer, *TNBC–NST* Triple-negative breast cancer of no special type

*Preoperative or †postoperative data retrieved from the Swedish National Register for Breast Cancer

Statistically significant differences (p 0.05) are highlighted in bold

### Association between histological type, age, and treatment

Sentinel lymph node biopsy, breast-conserving surgery, and adjuvant chemotherapy with anthracyclines were more frequently performed in patients with TNBC–NST, whereas those with TNBC–ILC more often underwent mastectomy and received adjuvant bisphosphonate therapy (Table 2). Use of neoadjuvant chemotherapy with an anthracycline and a taxane (~ 11%), adjuvant radiotherapy (~ 58%), and the rate of locoregional recurrence (~ 2%) were similar between the groups. Although distant relapse occurred more often among patients with TNBC–ILC, TNBC–NST was more commonly associated with late relapse or death occurring 2 years after diagnosis. Intriguingly, multivariable logistic regression showed that TNBC–ILC patients aged 40–49 years were 13 times more likely to receive a mastectomy and those 50–64 years were less likely to receive adjuvant chemotherapy (Table 3). Among women < 50 years, odds ratios were imprecise due to the small number of TNBC–ILC cases and these estimates should be interpreted with caution.

**Table 3:** Multivariable logistic regression analysis with odds ratio and 95% confidence intervals based on treatment modality and histological type

	Age group				
	< 40 years	40–49 years	50–64 years	65–75 years	≥ 75 years
	OR (95% CI)	OR (95% CI)	OR (95% CI)	OR (95% CI)	OR (95% CI)
BCS and ART vs other					
Histology					
TNBC–NST	1.00	1.00	1.00	1.00	1.00
TNBC–ILC	–	–	1.19 (0.60, 2.37)	0.76 (0.43, 1.35)	0.63 (0.20, 1.59)
Mastectomy vs other surgery					
Histology					
TNBC–NST	1.00	1.00	1.00	1.00	1.00
TNBC–ILC	–	**13.3 (1.77, 272)**	1.01 (0.48, 2.05)	1.10 (0.62, 1.94)	2.08 (0.92, 5.36)
ACT vs no ACT					
Histology					
TNBC–NST	1.00	1.00	1.00	1.00	1.00
TNBC–ILC	0.53 (0.049, 11.64)	0.87 (0.17, 5.02)	**0.43 (0.22, 0.83)**	1.01 (0.60, 1.73)	0.74 (0.28, 1.69)
NACT vs no NACT					
Histology					
TNBC–NST	1.00	1.00	1.00	1.00	1.00
TNBC–ILC	–	0.15 (0.0049, 1.47)	1.13 (0.45, 2.54)	0.88 (0.34, 1.99)	1.00 (0.22, 3.14)

*ACT* Adjuvant chemotherapy, *ART* Adjuvant radiotherapy, *BCS* Breast-conserving surgery, *CI* Confidence interval, *NACT* Neoadjuvant chemotherapy, *OR* Odds ratio, *TNBC* Triple-negative breast cancer, *TNBC–ILC* Triple-negative breast cancer–invasive lobular carcinoma, *TNBC–NST* Triple-negative breast cancer of no special type

Logistic regression models were adjusted for histological type, post-operative tumor size, tumor grade, and axillary lymph node status. Statistically significant models are shown in bold

### Association between histological type, age, and clinical outcome

**Fig. 3:**
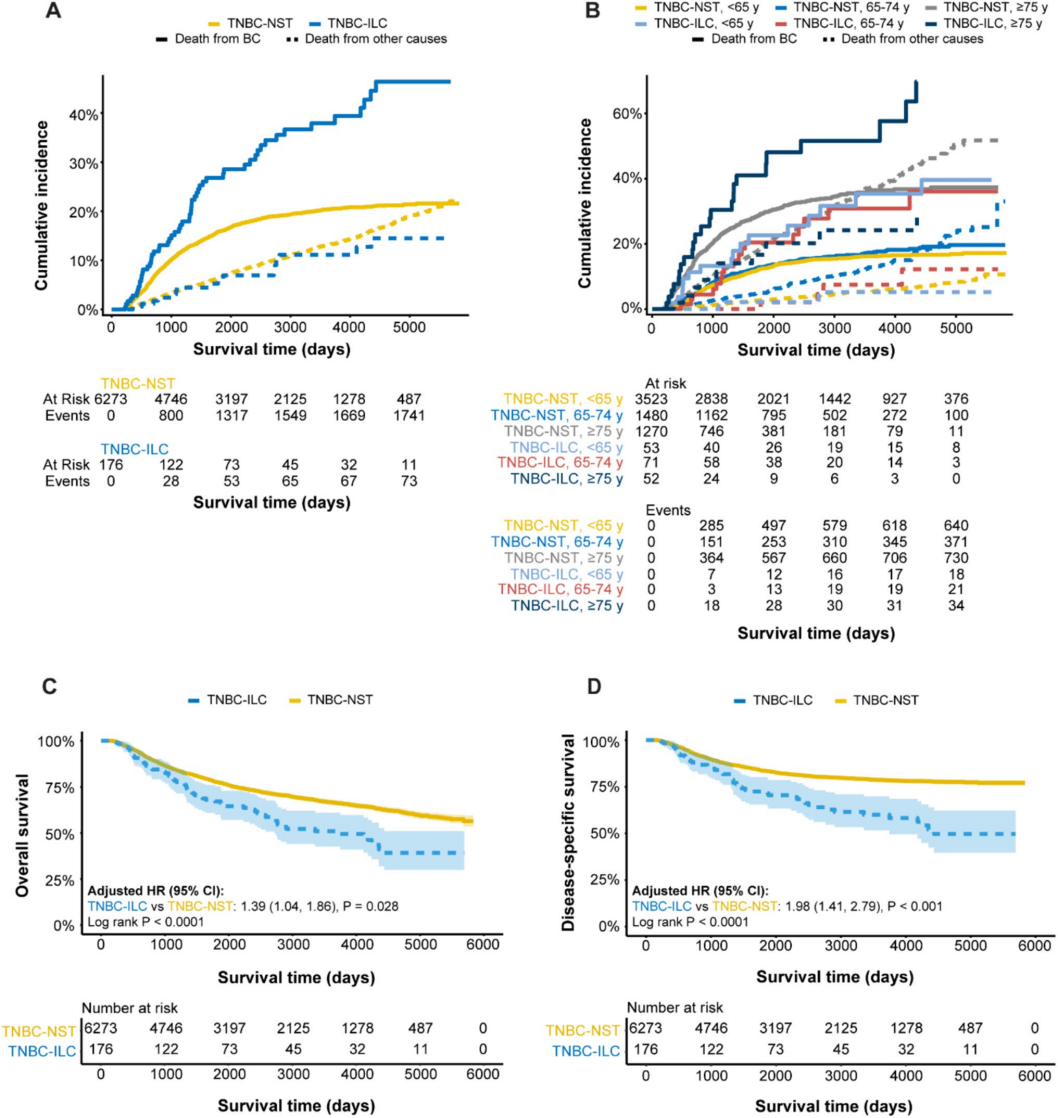
Survival analysis showing the relationship between histological type (TNBC invasive lobular carcinoma, TNBC–ILC; TNBC of no special type, TNBC–NST), age, and patient survival. Competing risk survival analysis of death from breast cancer (BC; solid line) or other causes (dotted line) in TNBC patients stratified by **A** histology and **B** histology and age. Patients with TNBC–ILC (aged 75 years and older) had the highest risk of death from breast cancer. By 8 years post-landmark time, death was attributable to other causes for women with TNBC–NST (aged 75 years and older). Kaplan–Meier analysis showing survival probabilities for **C** overall survival and **D** disease-specific survival based on histological type. Patients with TNBC-ILC had significantly more unfavorable prognoses. Multivariable Cox regression models were adjusted for patient age, tumor size, Nottingham histological grade (NHG), axillary lymph node status, CCIw group, and adjuvant chemotherapy received. Adjusted hazard ratios (HR) with 95% confidence interval (CI) and P-values calculated using the log-rank test are presented. The x-axes depict days after the landmark time and the y-axes depict survival probabilities. Shaded areas represent the 95% CI

An analysis of cumulative incidence demonstrated that TNBC–ILC, particularly those aged 75 years and older, was associated with the highest risk of death from breast cancer (Fig. 3). In contrast, TNBC–NST patients older than 75 years had a high risk of non-breast cancer-related death from 8 years post-landmark time. Kaplan–Meier estimates and multivariable Cox regression models confirmed these findings, thereby revealing that TNBC–ILC had the worst OS (log-rank P < 0.0001; adjusted HR 1.39 [95% CI 1.04, 1.86], P = 0.028) and DSS (log-rank P < 0.0001; adjusted HR 1.98 [95% CI 1.41, 2.79], P < 0.001), particularly for patients 50 years of age and older (Fig. S1). No differences in survival were found between the histological types for patients < 50 years of age. Although adjuvant chemotherapy was administered more often to patients with TNBC–NST, only this group showed a survival benefit from treatment (OS: log-rank P < 0.0001; DSS: log-rank P < 0.0001), suggesting limited responsiveness to adjuvant chemotherapy in TNBC–ILC (OS: log-rank P = 0.058; DSS: log-rank P = 0.49; Fig. S2). Overall, patients who received adjuvant chemotherapy had a 65% and 52% reduced hazard of OS and DSS over time than those not receiving treatment. As expected, 5-year survival probabilities were lowest for TNBC–ILC patients ≥ 75 years (OS: 42%; DSS: 55%) compared to those with TNBC–NST (OS: 51%; DSS: 68%; Table S1).

## Discussion

In this nationwide cohort of 6449 TNBC patients, TNBC–ILC represented a small yet distinct entity in contrast to TNBC–NST. TNBC–ILC patients were significantly older and more frequently ≥ 65 years of age, aligning with established data indicating that lobular histology typically presents later in life [13, 32]. Interestingly, despite the lower proliferative index and histologic grade typically associated with less aggressive disease, TNBC–ILC demonstrated a comparable frequency of lymph node involvement to TNBC–NST, suggesting that nodal spread may occur independently of these indolent features. The higher incidence of TNBC–ILC among older women is likely multifactorial, involving age-related accumulation of genetic alterations (e.g., *CDH1* mutations), immunosenescence, and the indolent, low-proliferative nature of lobular tumors. These factors collectively contribute to delayed onset and detection of ILC in postmenopausal women [7, 35].

Sociodemographic factors such as marital status and employment have been shown to influence both access to care and cancer outcomes, including breast cancer. Several studies have demonstrated that individuals who are unmarried, widowed, or divorced experience poorer cancer-specific survival, possibly due to reduced social support, delayed diagnosis, and lower likelihood of receiving guideline-concordant treatment [36–38]. Widowhood is particularly prevalent among older women, reflecting both gender differences in life expectancy and sociocultural patterns of marriage [37–44]. Similarly, unemployment or labor force withdrawal, especially among older adults, has been associated with lower socioeconomic status and reduced functional capacity, which may impact their ability to tolerate intensive cancer therapies [39–42]. In cancer registry datasets, individuals who are retired or on long-term medical leave are frequently categorized as unemployed or economically inactive, which could explain this discrepancy [45]. These indicators have therefore been proposed as potential markers of vulnerability, especially in patient subgroups with complex disease profiles or high comorbidity burden [43]. Incorporating sociodemographic variables into the interpretation of clinical outcomes may be particularly important in rare breast cancer subtypes such as TNBC–ILC.

Moreover, the higher comorbidity burden observed among TNBC–ILC patients, including secondary malignancies, may further limit occupational engagement and functional independence. These sociodemographic factors may also interact with clinical outcomes. Comorbid conditions, especially other malignancies, were present in 47% of TNBC–ILC patients, consistent with previous observations that comorbidity drives less aggressive treatment and poorer survival [46, 47] and may further reflect the cumulative burden of aging, earlier treatments and lower socioeconomic status.

TNBC–ILCs typically exhibit apocrine morphology, E-cadherin negativity, androgen receptor positivity, and high histological grade [28, 48]. In the present study, clinicopathological assessment revealed that TNBC–ILCs were more often T3 with lower proliferative index (Ki-67 < 20%) and less often histological grade 3, characteristics atypical for TNBC but consistent with lobular biology [19, 49, 50], challenging the conventional association of TNBC with high-grade, highly proliferative tumors. While these features might suggest a less aggressive phenotype, survival analyses contradicted this assumption. Paradoxically, TNBC–ILC exhibited worse survival, emphasizing that less aggressive histologic features did not translate into better outcomes, especially in patients ≥ 50 years. Although prior studies have reported poorer clinical outcomes for patients with TNBC–ILC compared to those with TNBC–NST and hormone receptor-positive ILC (HR-ILC) [48, 51, 52], Joshi et al. found that the adjusted overall survival for TNBC-ILC did not differ significantly from that of TNBC–NST [18]. Despite growing concern that patients with TNBC–ILC may be under-treated due to diagnostic challenges or comorbidities [31, 53], studies on TNBC–ILC remain underrepresented due to its low incidence [52, 54–56].

Treatment disparities were prominent. Interestingly, younger TNBC–ILC patients aged 40–49 years were 13 times more likely to receive a mastectomy compared to TNBC-NST patients. the patient in age 50–64 years were less likely to receive adjuvant chemotherapy compared to same age in TNBC-NST group. This age-specific variation aligns with broader literature showing de-escalation of systemic therapy in elderly TNBC patients due to age-related concerns, comorbidity burden, and frailty [57] and possibly also physician perceptions of tumor biology in TNBC–ILC. In a systematic review, Yoon et al. noted significantly lower chemotherapy use in patients ≥ 70 years, often tied to reduced performance status [57]. These treatment disparities in our study and in TNBC–ILC, particularly the reduced use of adjuvant chemotherapy among patients aged 50–64, may in part reflect a history of prior cancer treatments. Previous malignancies and their associated therapies (e.g., chemotherapy, radiation, or targeted agents) can limit tolerance for further systemic treatment due to cumulative toxicity, organ dysfunction, or increased frailty. This is consistent with evidence suggesting that a history of prior cancer influences treatment decisions and is associated with undertreatment in subsequent malignancies [32, 58]. Although TNBC is generally treated aggressively due to its poor prognosis, the atypical features of TNBC–ILC may lead to less standard management, possibly contributing to the observed survival disadvantage [59].

Additionally, machine learning feature selection confirmed age and post-operative tumor size as the most predictive variables for identifying TNBC–ILC. This reinforces the importance of considering age- and subtype-specific treatment strategies, particularly in older patients, in whom competing risks of non-cancer-related mortality are also elevated and should support the notion that demographic and tumor burden factors should guide risk stratification and management. Finally, TNBC–ILC patients older than 75 years of age had the poorest 5-year OS (42%) and DSS (55%), whereas older TNBC–NST patients mainly succumbed to non-breast cancer causes after 8 years. This reflects the dual hazards of cancer-specific mortality and competing comorbidities [32]. Failure to deliver standard-of-care adjuvant therapies likely contributes to the unfavorable DSS seen in older TNBC–ILC cases [32, 60–62]. Modeling these outcomes into clinical practice, our data reinforce that TNBC–ILC is not simply a histologic rarity but a high-risk subtype that disproportionately affects older, demographically vulnerable individuals with complex treatment needs.

The strengths of this study include its nationwide, population-based design, which minimizes selection bias and ensures representativeness, as well as access to high-quality Swedish registry data with long follow-up. Despite the rarity of TNBC–ILC, this study comprises one of the largest cohorts to date, allowing meaningful comparisons with TNBC–NST. The integration of sociodemographic variables and the use of both classical statistical and machine learning approaches further add to the novelty and robustness of our findings. Limitations include the retrospective design, the relatively small number of TNBC–ILC cases, and incomplete recurrence data from some regions. The very wide confidence intervals in age groups < 50 years (TNBC–ILC) reflect sparse data rather than a large true effect size. Therefore, these subgroup results are exploratory. Detailed treatment information was not available, and socioeconomic variables may have been subject to misclassification. In addition, histological misclassification cannot be excluded, and molecular profiling beyond standard receptor status was lacking. Finally, generalizability to non-Swedish healthcare systems may be limited.

Our study offers several entirely new insights, specifically in socioeconomic disparities, age-dependent treatment decisions, adjuvant bisphosphonate usage, machine learning-based predictors, and detailed stratified survival data. These contributions significantly advance our understanding of TNBC–ILC beyond previously reported clinicopathologic features. Taken together, our findings underscore the importance of recognizing TNBC–ILC as a distinct clinical entity with unique demographic, pathological, and prognostic characteristics. Further research is needed to optimize treatment strategies and improve outcomes in this vulnerable subgroup.

## Conclusions

This study highlights that TNBC–ILC is a rare but prognostically distinct subtype, affecting older women who are more likely to be widowed, unemployed, and carry comorbidities including previous malignancies. Despite presenting with lower-grade, slower-proliferating tumors, TNBC–ILC is associated with worse disease-specific survival, especially in patients ≥ 50 years. The lower rates of adjuvant chemotherapy and the variation in surgical approaches observed among older patients likely reflect treatment de-escalation driven by age-related factors, earlier chemotherapy, and comorbidities. These findings underscore the need for subtype- and age-tailored treatment algorithms, integrating comprehensive geriatric assessment to ensure that biologic features, not just age and frailty, inform adjuvant therapy decisions. Further prospective studies or registry data are warranted to refine treatment guidelines, particularly in the older TNBC–ILC cohort, and to evaluate the impact of complete multimodal therapy on disease outcomes.

## Supplementary Information

Below is the link to the electronic supplementary material.

Supplementary file1 (PDF 666 KB)

## Data Availability

Requests for sharing of de-identified individual participant data and a data dictionary defining each field in the set will be considered by the corresponding author.
