# Supplementary material for: Worse survival despite indolent features for triple-negative invasive lobular carcinoma: a Swedish nationwide registry-based study
Source: Breast Cancer Res Treat. 2025 Nov 21;215(1):12. doi: 10.1007/s10549-025-07862-9 (PMC12638390; doi:10.1007/s10549-025-07862-9)
Supplement: Supplementary file 1 — Supplementary file1 (PDF 666 KB) [file 10549_2025_7862_MOESM1_ESM.pdf]

Supplementary material

Worse survival despite indolent features for triple-negative invasive lobular carcinoma: A Swedish nationwide registry-based study

Jenny Nyqvist-Streng, Chaido Chamalidou, Anikó Kovács, Toshima Z. Parris

Contents

Figure S1 3
Figure S2 4
Table S1 5

Figure S1

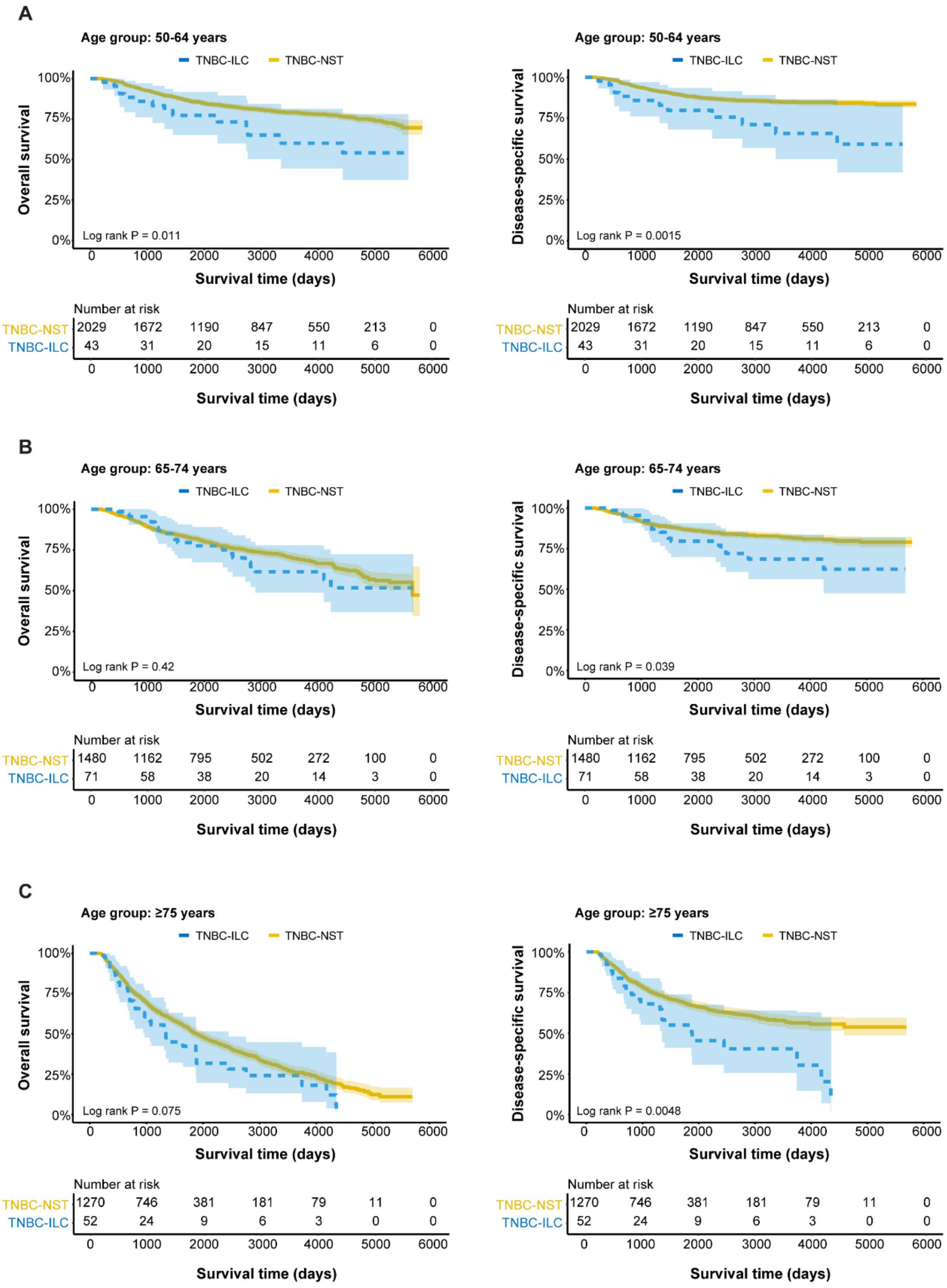

Supplementary Fig. 1. Estimates of the probability of overall survival and disease-specific survival based on histological type (TNBC-ILC, triple-negative breast cancer invasive lobular carcinoma; TNBC-NST, triple-negative breast cancer of no special type) and patient age. Patients with TNBC-ILC (>50 years) had significantly more unfavorable prognoses. p-values were calculated using the log-rank test. The x-axes depict days after landmark time and the y-axes depict survival probabilities. Shaded areas represent the 95% confidence intervals.

Figure S2

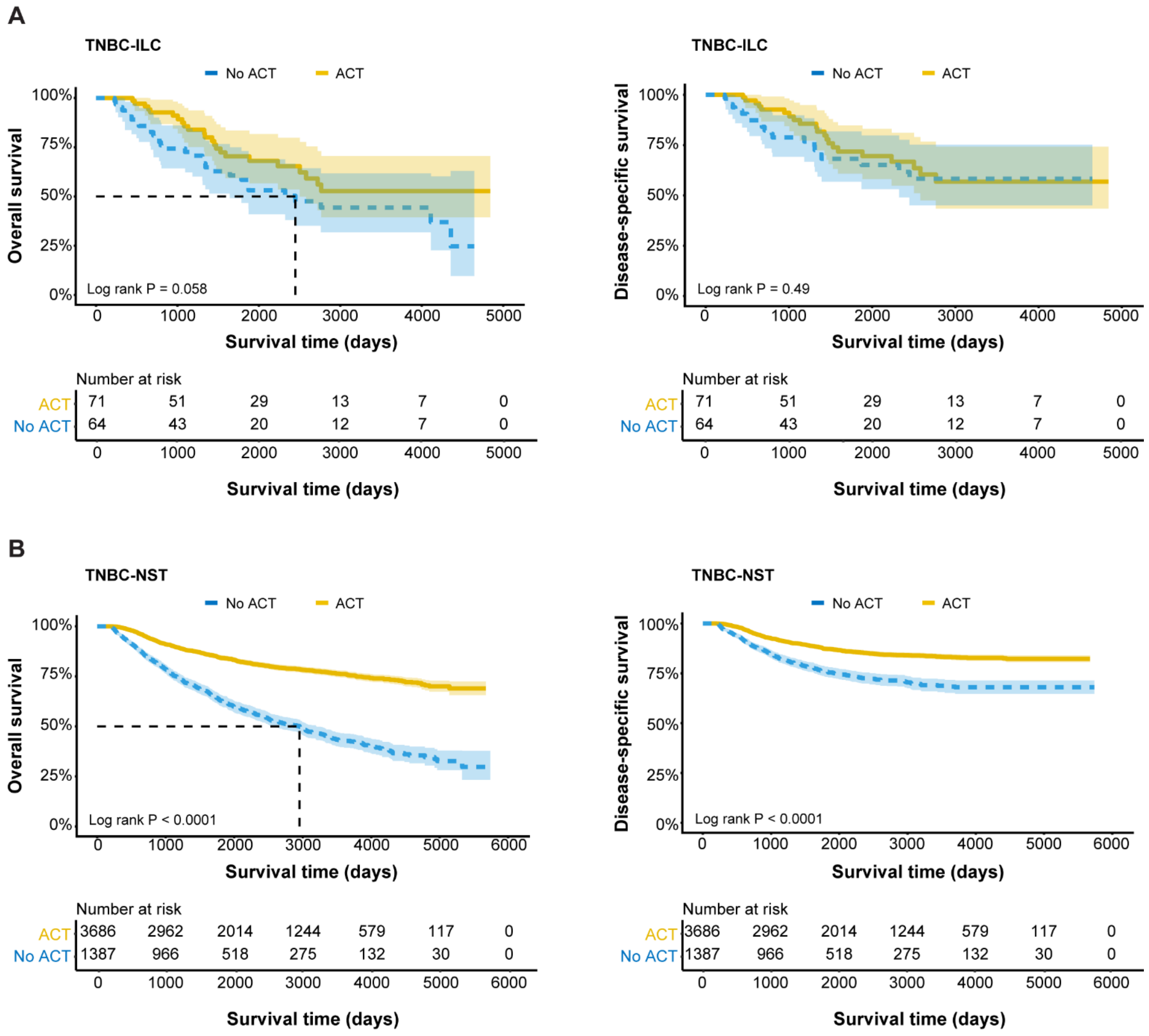

Supplementary Fig. 2. Estimates of the probability of overall survival and disease-specific survival based on histological type (**A** TNBC-ILC, triple-negative breast cancer invasive lobular carcinoma; **B** TNBC-NST, triple-negative breast cancer of no special type) and adjuvant chemotherapy (ACT) administration. Patients with TNBC-NST not receiving ACT had significantly more unfavorable prognoses, while no survival benefit of treatment was found for those with TNBC-ILC. p-values were calculated using the log-rank test. The x-axes depict days after landmark time and the y-axes depict survival probabilities. Shaded areas represent the 95% confidence intervals.

Table S1

Percentages for 1-, 3-, and 5-year survival probabilities for overall survival and disease-specific survival, stratified by histological type

	1 year (95% CI)		3 year (95% CI)		5 year (95% CI)	
**Age groups**	**TNBC-ILC**	**TNBC-NST**	**TNBC-ILC**	**TNBC-NST**	**TNBC-ILC**	**TNBC-NST**
OS						
All patients	97 (93.9, 99.3)	97 (97.0, 97.8)	80 (73.7, 86.2)	85 (84.3, 86.1)	67 (59.9, 75.2)	77 (76.3, 78.6)
<40 years	100 (100, 100)	99 (98.3, 99.9)	100 (100, 100)	90 (86.2, 91.7)	67 (30.0, 100)	83 (79.5, 86.4)
40-49 years	100 (100, 100)	99 (98.0, 99.5)	86 (63.3, 100)	89 (87.3, 91.3)	71 (44.7, 100)	84 (81.4, 86.4)
50-64 years	98 (93.3, 100)	99 (98.7, 99.5)	83 (72.6, 95.4)	92 (90.5, 93.0)	77 (65.1, 91.7)	86 (84.1, 87.4)
65-74 years	100 (100, 100)	98 (97.2, 98.6)	92 (85.5, 99.0)	88 (86.4, 89.8)	78 (67.4, 89.2)	82 (80.0, 84.2)
≥75 years	90 (82.7, 98.8)	93 (91.1, 94.0)	56 (42.6, 72.6)	67 (63.8, 69.2)	42 (29.6, 60.6)	51 (48.0, 54.0)
DSS						
All patients	97 (94.7, 99.6)	98 (97.7, 98.4)	84 (78.5, 89.9)	89 (87.9, 89.6)	72 (65.5, 80.1)	84 (82.6, 84.6)
<40 years	100 (100, 100)	99 (98.8, 100)	100 (100, 100)	91 (88.1, 93.3)	67 (30.0, 100)	85 (81.7, 88.3)
40-49 years	100 (100, 100)	99 (98.3, 99.6)	86 (63.3, 100)	91 (88.7, 92.5)	71 (44.7, 100)	87 (84.1, 88.8)
50-64 years	98 (93.3, 100)	99 (98.9, 99.6)	86 (76.2, 97.0)	93 (91.8, 94.1)	80 (68.1, 93.6)	88 (86.9, 89.9)
65-74 years	100 (100, 100)	98 (97.7, 99.0)	92 (85.8, 99.0)	91 (89.3, 92.3)	80 (69.8, 90.7)	87 (84.9, 88.6)
≥75 years	92 (85.3, 99.8)	95 (93.3, 95.8)	68 (55.2, 83.8)	77 (74.2, 79.2)	55 (41.0, 73.9)	68 (65.0, 70.9)

Abbreviations: DSS, Disease-specific survival; TNBC, Triple-negative breast cancer; TNBC-ILC, Triple-negative breast cancer invasive lobular carcinoma; TNBC-NST, Triple-negative breast cancer of no special type; OS, Overall survival
